# Mitigating Disputes Originated by Multiple Discordant Systematic Reviews and Meta-Analyses: A Survey of Methodologists and Clinicians

**DOI:** 10.3389/frma.2022.849019

**Published:** 2022-04-15

**Authors:** Livia Puljak, Elena Parmelli, Matteo Capobussi, Marien Gonzalez-Lorenzo, Alessandro Squizzato, Lorenzo Moja, Nicoletta Riva

**Affiliations:** ^1^Center for Evidence-Based Medicine and Health Care, Catholic University of Croatia, Zagreb, Croatia; ^2^Department of Epidemiology, Lazio Region-ASL Roma 1, Rome, Italy; ^3^Department of Biomedical Sciences for Health, University of Milan, Milan, Italy; ^4^Laboratory of Clinical Research Methodology, Istituto di Ricerche Farmacologiche Mario Negri IRCCS (Istituto di Ricovero e Cura a Carattere Scientifico), Milan, Italy; ^5^Department of Medicine and Surgery, Research Center on Thromboembolic Disorders and Antithrombotic Therapies, University of Insubria, Varese, Italy; ^6^Department of Pathology, Faculty of Medicine and Surgery, University of Malta, Msida, Malta

**Keywords:** systematic review, overview of systematic review, certainty of evidence, summary of findings table, discordant evidence

## Abstract

**Background:**

Overlapping systematic reviews (SRs) are increasingly frequent in the medical literature. They can easily generate discordant evidence, as estimates of effect sizes and their interpretation might differ from one source to another.

**Objective:**

To analyze how methodologists and clinicians make a decision when faced with discordant evidence formalized in structured tables.

**Methods:**

We conducted a 16-item survey exploring how methodologists and clinicians would react when presented with multiple Summary of Findings (SoF) tables (generated using the GRADE tool) derived from 4 overlapping and discordant SRs and meta-analyses on thrombolytic therapy for intermediate-risk pulmonary embolism. SoF tables reported 4 different magnitudes of effects and overall certainty. Participants were asked to provide their recommendations regarding the intervention and the reasons behind their conclusion.

**Results:**

Of the 80 invitees, 41 (51%) participated. The majority described themselves as “somewhat familiar” or experts with SoF tables. The majority recommended the therapy (pharmacological systemic thrombolysis), grading the recommendation as weak positive. Certainty of evidence and benefit-risk balance were the two criteria that prevailed in generating the recommendation. When faced with overlapping meta-analyses, the preferred approach was to use only high-quality SRs and exclude redundant SRs. Several participants suggested integrating the SoF tables with additional information, such as a more comprehensive evaluation of the risk of bias of systematic reviews (71%), heterogeneity/inconsistency (68%) and studies included within each SR (62%).

**Conclusion:**

When faced with multiple controversial SR results, the type and completeness of reported information in SoF tables affect experts' ability to make recommendations. Developers of the SoF table should consider collating key information from overlapping and potentially discordant reviews.

## Introduction

In 1992 evidence-based medicine (EBM) was pronounced as a new approach for teaching and practicing clinical medicine (Evidence-Based Medicine Working Group, 1992). In the past 25 years, the EBM movement succeeded in promoting rigorous and explicit methods to identify, select and appraise relevant research studies. Systematic reviews (SRs), meta-analyses (MAs), clinical practice guidelines, health technology assessment and, more recently, overviews of systematic reviews (OSRs) are evidence synthesis designs that gained increasing popularity. SRs and MAs play a pivotal role, as they often inform other evidence synthesis designs. Guidelines, health technology assessment and OSRs are built systematically from the lower levels of evidence and provide substantially more useful information for guiding clinical decision-making (Alper and Haynes, 2016). For instance, many systematically derived recommendations rely on SRs that were either previously published or created *de novo* by guideline developers (Cook et al., 1997).

The number of SRs and MAs is increasing. For example, Hoffmann and colleagues observed a more than 20-fold increase in the number of SRs indexed on PubMed between 1990 and 2019 (Hoffmann et al., 2021). It has been suggested that the increasing number of redundant, unnecessary and misleading evidence syntheses, described by the claim “too much evidence,” is one of the reasons for a potential crisis of the EBM movement (Greenhalgh et al., 2014).

OSRs, as the next-generation overarching study design, have been proposed as a solution for the limited utility of multiple SRs (Ioannidis, 2017). OSRs are studies that allow synthesizing a large amount of evidence from multiple SRs and identifying gaps, weaknesses and biases that affect certain research fields (Puljak, 2017). However, one of the problems that OSRs can detect is the existence of discordant SRs, i.e., reviews that cover the same topic but reach different results and different conclusions (Riva et al., 2018; Augustin et al., 2020).

Summary of Findings (SoF) tables have been designed to summarize the key results of an SR or MA and evaluate the authors' confidence in the estimates of effect in evidence syntheses (Guyatt et al., 2011). However, it is unknown which information methodologists and clinicians prioritize when faced with discordant evidence from multiple SRs or MAs, and how their decision-making algorithm could be used to improve SoFs.

This study aimed to analyze how methodologists and clinicians decide when faced with discordant evidence and how the SoF table should be constructed in the event of overlapping MAs.

## Methods

### Ethics

The study protocol was approved by the Ethics Committee of the University of Split School of Medicine (Approval no. 2181-198-03-04-17-0064).

### Study Design

This was a cross-sectional study.

### Participants

This study was conducted during the year 2017 among clinicians considered opinion-leaders in the field of pulmonary embolism and methodologists skilled in EBM. We were targeting researchers with a high number of articles (i.e., more than 10) published on topics related to this survey (i.e., pulmonary embolism, systematic, reviews, SoF tables) and a high number of citations (i.e., more than 1,000), to ensure that these individuals are recognized experts in the field. Participants were recruited by using the snowballing method.

### Discordant Evidence Scored by the Participants

We invited participants to complete a scenario-based survey. The participants were asked to identify the source of discordance between contrasting evidence on pharmacological systemic thrombolytic therapy for intermediate-risk pulmonary embolism and suggest how to incorporate them in developing recommendations. They were also asked to provide suggestions for an accurate and transparent reporting of legitimate controversies in the final guideline.

For this study, we prepared four SoF tables (Supplementary File 1) from our OSR about thrombolytic therapy for intermediate-risk pulmonary embolism using the Grading of Recommendations Assessment, Development and Evaluation (GRADE) tool (Riva et al., 2018). We chose four SRs, purposively creating a sample of real SRs with different salient characteristics. Namely, we included the SR with the highest AMSTAR (A MeaSurement Tool to Assess systematic Reviews) score (Hao et al., 2015), the SR with the lowest AMSTAR score (Gao et al., 2015), the SR with the highest number of included trials (Chen and Ren, 2014) and the SR with the highest number of citations (Chatterjee et al., 2014). Four authors produced these SoF tables (LM, EP, NR, LP) in a way that each SoF was created independently by two authors, and then the results were compared and discrepancies discussed until they were resolved. Authors resolved discrepancies *via* discussion. Respondents did not receive information about the SRs from which the SoF tables were extracted.

### Survey

We created a 16-item survey (available in Supplementary File 2) divided into three sections for this study. In the first section, there were questions about characteristics of the participants, including their sex, age, place of residence, personal description of their role and familiarity with an SoF table. In the second section, the participants were asked to make a decision based on the four presented SoFs, explaining how they reached their decision when faced with discordant evidence and how they would judge the quality of presented evidence. In the third section, there were general questions about decision-making in the event of discordant evidence from overlapping MAs and how SoF tables should be designed in the event of overlapping MAs.

### Dissemination of the Survey

Prospective participants were invited by e-mail to participate in the study, which was available as an online survey at the SurveyMonkey platform (SurveyMonkey Inc., Palo Alto, CA, USA). The survey was set up as completely anonymous; it did not collect participants' internet protocol (IP) addresses. Participants received a maximum of 3 reminders before the survey was closed.

### Statistics

Descriptive statistics with frequencies and percentages were used based on the built-in statistical features of SurveyMonkey. Characteristics of clinicians vs. methodologists were compared using the Chi Square or the Fisher's exact tests, as appropriate. The software STATA/BE version 17 (StataCorp LP, College Station, TX) was used for statistical analysis with two-tailed *p* < 0.05 considered statistically significant.

## Results

Of the 80 invited participants, 41 (51%) accepted the invitation and participated in the study. A similar number of women (*N* = 20; 49%) and men participated. Most of the participants were aged 41 to 50. Most had a residence in Europe (29, 71%), and most of them were either methodologists (*N* = 22; 54%) or clinicians (*N* = 15; 37%). The majority of participants described themselves as ‘somewhat familiar' with the SoF tables (*N* = 15; 37%) or experts involved in GRADE methods and who can teach others about creating the SoF tables (*N* = 12; 29%) (Table 1).

**Table 1:** Characteristics of study participants (responses provided by all 41 participants).

**Characteristic**	*N* (%)^*^
**Sex**	
Women	20 (49)
Men	21 (51)
**Age**	
Up to 40 years	11 (21)
41–50 years	17 (42)
51 years or older	13 (32)
**Place of work**	
Asia	1 (2.4)
Australia	1 (2.4)
Europe	31 (76)
North America	8 (20)
**Personal description of their role**	
Clinician	15 (37)
Methodologist	22 (54)
Other	4 (10)
**How familiar are you with summary of findings (SoF) tables?**	
Not familiar at all (I've never used them)	1 (2.4)
Very little familiar (I've only used them once)	3 (7.3)
A bit familiar (I've used them more than once, but I still need help when creating one)	3 (7.3)
Somewhat familiar (I've used them on several occasions but still need help on some issues)	15 (37)
Very familiar (I've used them on several occasions and/or I can help others create them)	7 (17)
Expert (I am involved in GRADE methods and I can teach others how to create SoF tables)	12 (29)

* *The percentages may not add up to 100 due to rounding*.

Based on the evidence/SoF tables presented, most participants proposed that thrombolytic therapy (i.e., infusion into a vein of a medicine that will break up or dissolve blood clots, in addition to an anticoagulant medicine) “is suggested” or “might be suggested” in patients with pulmonary embolism at intermediate-risk of death at 30 days. They also mostly agreed that the grade for this recommendation was weak positive. Most of them (*N* = 27; 79%) based their recommendation on two or more SoF tables. They identified the most important outcome (i.e., mortality) and then considered that the quality of evidence for this outcome was marked in the preferred SoF tables as “high.” They reported that the certainty of the evidence and the benefit-risk balance were two criteria that most often guided their choice of recommendation (Table 2).

**Table 2:** Participants' recommendation regarding the intervention, and rationale behind their decision-making (responses provided by 34 participants).

**Question**	***N* (%)**
**Based on this evidence, please provide your recommendation: “in patients with intermediate risk pulmonary embolism, thrombolytic therapy compared with anticoagulation alone is”**	
Recommended	4 (12)
Suggested	11 (32)
Might be suggested	15 (44)
Not recommended	4 (12)
**The grade for this recommendation is:**	
Strong positive	5 (15)
Weak positive	22 (65)
Weak negative	7 (21)
Strong negative	0
**Which summary of findings table guided your decision?**	
Summary of findings table n. 1	0
Summary of findings table n. 2	0
Summary of findings table n. 3	5 (15)
Summary of findings table n. 4	2 (6)
Two or more summaries of findings table	27 (79)
None	0
**The quality of evidence of the outcome that you consider to be the most important is:**	
High	19 (59)
Moderate	10 (29)
Low	5 (15)
Very low	0
**Which criteria related to the summary of findings table guided your choice? (multiple answers allowed)**	
N° of studies included	7 (21)
N° of patients included	14 (41)
Quality of the evidence	28 (82)
The benefit-to-risk ratio	28 (82)
Supplementary analyses (e.g., sensitivity, trial sequential analysis mentioned in summary of findings 2)	11 (32)
Evidence published in languages other than English	2 (6)
My personal knowledge of the literature in the field or experience (criterion not related to the Summary of Findings)	4 (12)

**Figure 1:**
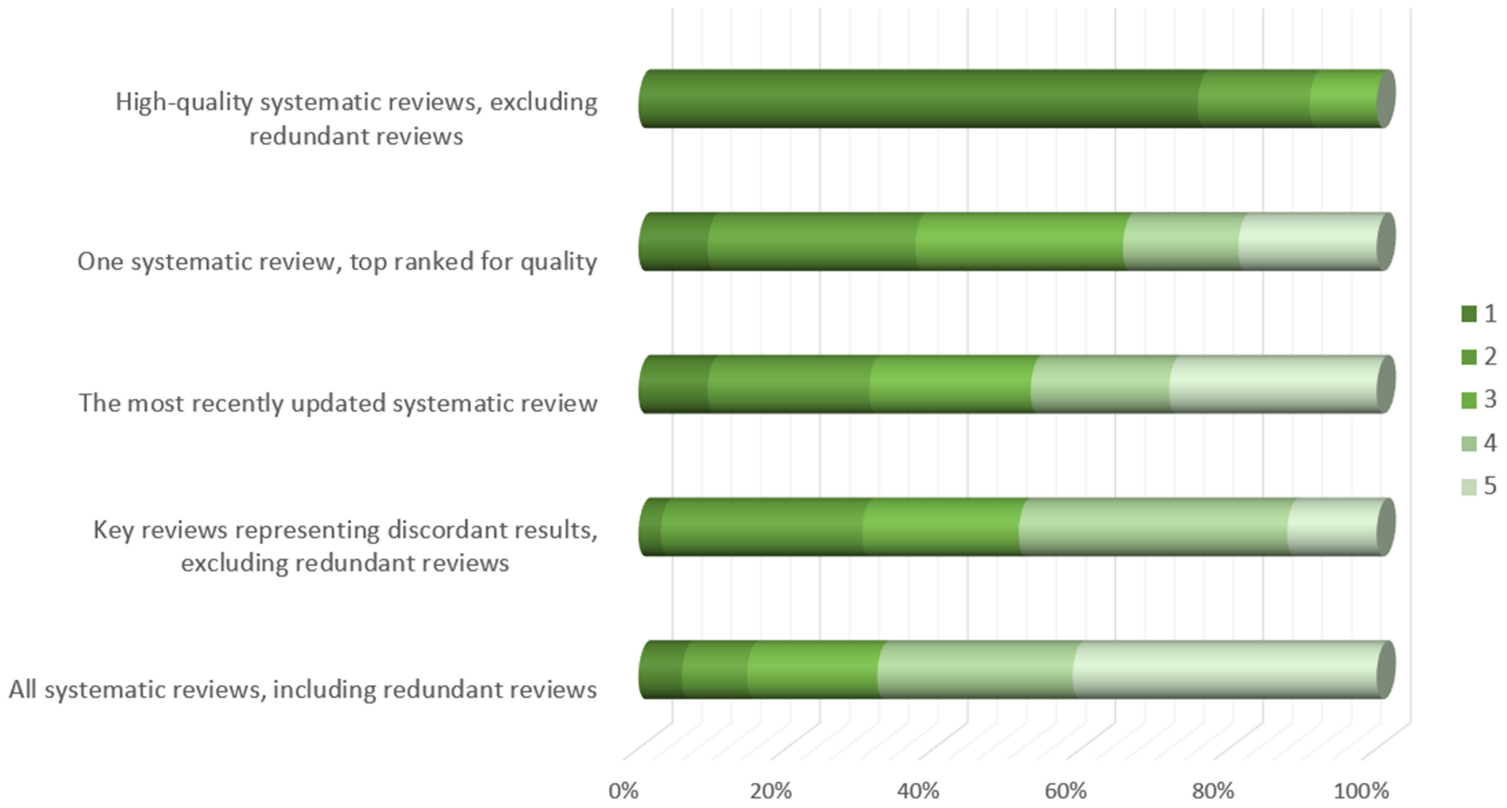
Participants' opinion about approaches to be taken for supporting guideline development group decisions in case of overlapping meta-analyses (responses provided by 34 participants). Figure shows responses to the following question: Overlapping meta-analyses can often be confusing because they may reach different conclusions. In such cases, which approach could be the most effective in supporting guideline development group decisions? Please order the statements by relevance, the most relevant = 1.

When faced with overlapping MAs, which may reach different conclusions, the participants suggested using only high-quality SRs and excluding redundant reviews as the most effective strategy in supporting guideline development (*N* = 25; 76% of the participants gave this option the highest priority mark) (Figure 1).

**Figure 2:**
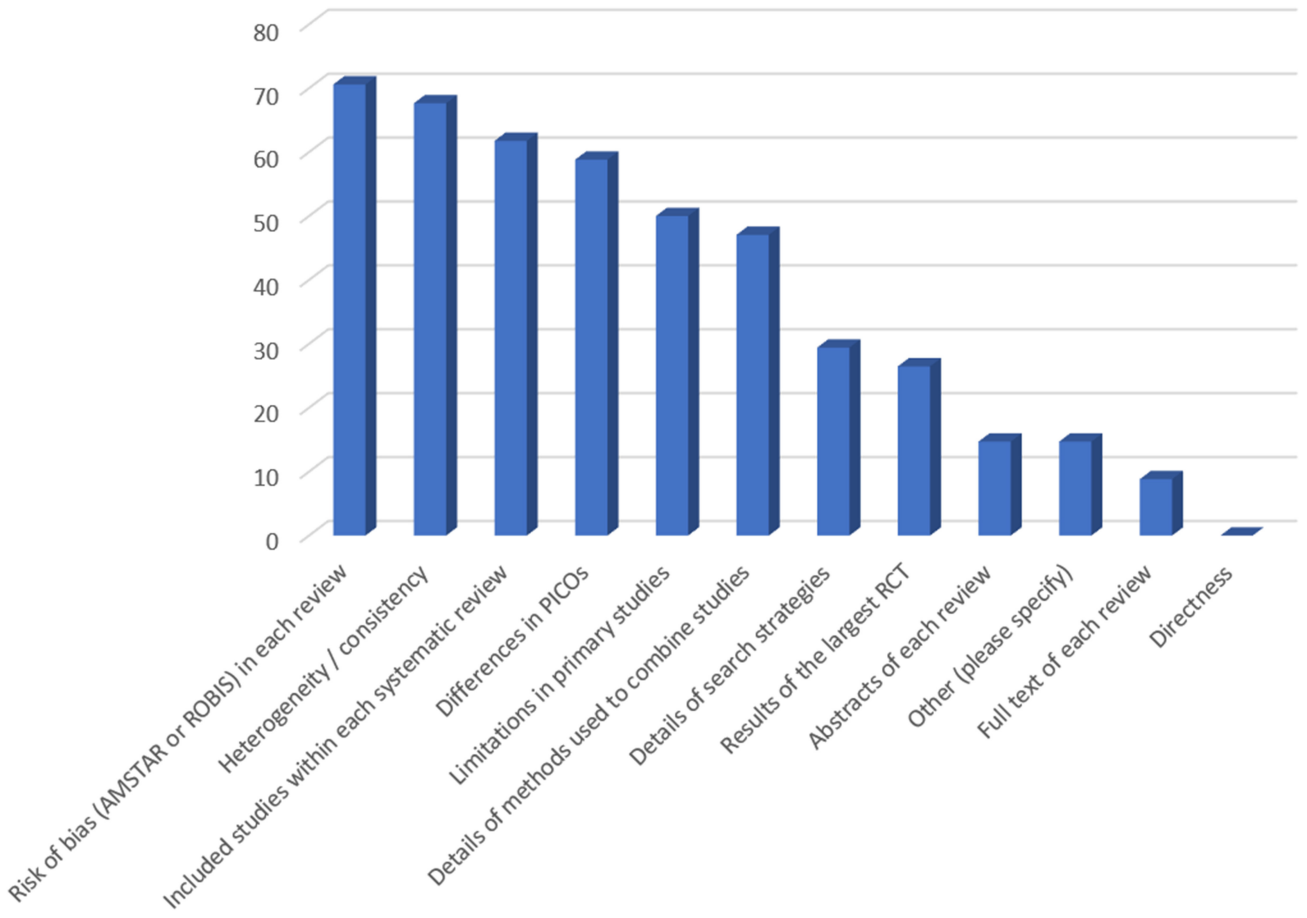
Suggestions of participants regarding information needed for decision-making in case of overlapping systematic reviews (responses provided by 34 participants). Figure shows responses to the following question: In case of overlapping meta-analyses, on top of summary of findings table, which additional information would you like to have? (multiple answers allowed). Y-axis denotes percentage of participants.

**Figure 3:**
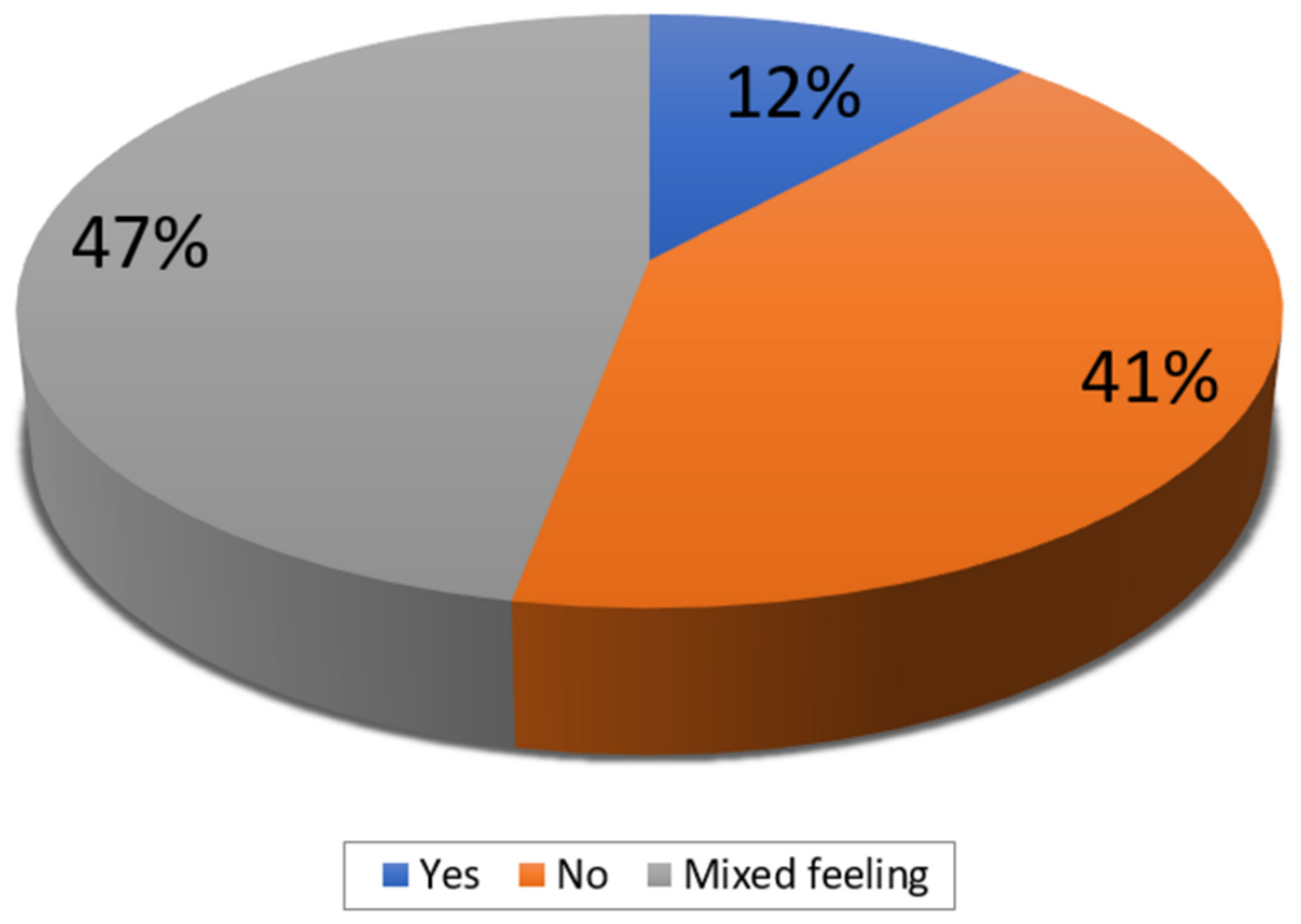
The participants' opinion on whether the actual form of the summary of findings table captures differences across overlapping systematic reviews (responses provided by 34 participants).

In case of overlapping MAs, in addition to the SoFs, additional information that the participants would like to have were: 1. the risk of bias in each review (measured with AMSTAR or a tool for assessing the risk of bias in systematic reviews—ROBIS) (*N* = 24; 71%), 2. the heterogeneity / consistency among the results of included studies/SRs (*N* = 23; 68%), and 3. the included studies within each SR (*N* = 21; 62%) (Figure 2). In addition, 41% of the participants indicated that they do not think that the actual form of the SoF table supports users in capturing differences across overlapping SRs (Figure 3). However, a minority did not consider overlapping SRs a problem deserving a dedicated approach in GRADE. Some participants indicated that it is not possible to improve the SoF to present evidence from overlapping reviews, mainly because clinical decisions should be based on one SR, which is the most up-to-date, or the one with the highest quality, accompanied by a clear justification for this choice (Table 3).

**Table 3:** Categorized suggestions to revise the summary of findings template in case of overlapping reviews (open-ended response).

**Specific suggestions provided by participants for improving the SoF template in case of overlapping reviews:**	1. Provide more details about reasons for discordant results:	a. Provide link to each included article; provide reference lists of included studies in each review; identification of included studies to make it easier to identify reasons for variations; need citations to quickly see which RCTs overlap	b. Add review year of publication/period covered by the search strategy; date of search of review	c. Provide more detailed PICO (such as, definition of intermediate risk of mortality and inclusion criteria in this case); report PICO	d. Report search strategy	e. Report pooling methods	f. Reporting rigor of criteria for judging risk of bias of the same primary studies; evaluation of risk of bias of each article in order to compare this evaluation across reviews	g. Reporting reasons for discordant results	h. The number of studies overlapping all reviews	2. Add NNT/NNH	3. There should be one table that will make comparison easier, with a link to the other SoFs or pop-up box with data from the other SoFs	4. Only updated and high-quality reviews should be presented	5. ROBIS of review	6. SoF could provide “sensitivity” analyses, e.g., based on study quality vs. exhaustive review	**Opinion regarding barriers/impossibility to fix the SoF; or SoF is irrelevant for decision making:**	7. Difficult to do, as they do not capture difference in primary studies, but assume their homogeneity	8. No way to improve the SoFs, differences need to be assessed at the level of the SR. In this scenario I would like to access the SRs that generated the SoF tables	9. Those creating clinical practice guidelines need to base their recommendations on SRs—to identify the most up-to-date high-quality SR and use GRADE to assess the body of evidence, and if there are several SRs than developers need to provide rationale for picking a particular SR, but I am not sure that this belongs in the SoF table	10. No way to improve the SoF, one should either choose one or more high quality SR, or do another, better one, yourself

*Responses provided by 19 participants*.

*GRADE, Grading of Recommendations Assessment; Development and Evaluation; NNH, number needed to harm; NNT, number needed to treat; PICO, Participants; Intervention; Comparator; Outcomes (model for asking focused clinical questions); RCT, randomized controlled trial; ROBIS, A Risk of Bias Assessment Tool for Systematic Reviews; SoF, Summary of Findings table*.

An open-ended question about ideas to improve the SoF in case of overlapping SRs yielded 19 responses (Table 3). Participants mostly provided suggestions for improving the SoF table by having the option to present the results of the overlapping SRs. Most of them suggested that various information should be added to the SoF, which may help in discerning reasons for discrepancies.

When we compared responses between clinicians and methodologists, we found that methodologists were more often experts or very familiar with the SoF. There were no significant differences in the type of recommendation these two groups provided based on the evidence presented and their grade of recommendation (Table 4). There was no difference in the frequency of the chosen criteria related to the SoF table, which guided the decision making. Methodologists more commonly indicated that they would need detailed information on the included studies within each systematic review to make a decision (Table 4).

**Table 4:** Differences in responses between clinicians and methodologists.

	**Clinicians**	**Methodologists**	***P*-value**
**Sex**, ***n*** **(%)**			0.004
Women	3 (20.0%)	15 (68.2%)	
Men	12 (80.0%)	7 (31.8%)	
**Age**, ***n*** **(%)**			0.577
Up to 40 years	3 (20.0%)	5 (22.7%)	
41–50 years	8 (53.3%)	8 (36.4%)	
51 years or older	4 (26.7%)	9 (40.9%)	
**Place of work**, ***n*** **(%)**			0.896
Asia	0 (0%)	1 (4.6%)	
Australia	0 (0%)	1 (4.6%)	
Europe	11 (73.3%)	16 (72.7%)	
North America	4 (26.7%)	4 (18.2%)	
**Knowledge of the summary of findings tables**, ***n*** **(%)**			0.006
Not familiar at all	1 (6.7%)	0 (0%)	
Very little familiar	2 (13.3%)	1 (4.6%)	
A bit familiar	1 (6.7%)	1 (4.6%)	
Somewhat familiar	7 (46.7%)	6 (27.3%)	
Very familiar	4 (26.7%)	3 (13.6%)	
Expert	0 (0%)	11 (50.0%)	
**Provided recommendation: “in patients with intermediate risk pulmonary embolism, thrombolytic therapy compared with anticoagulation alone is,”** ***n*** **(%)**			0.171
Recommended	1 (7.1%)	3 (17.7%)	
Suggested	3 (21.4%)	7 (41.2%)	
Might be suggested	9 (64.3%)	4 (23.5%)	
Not recommended	1 (7.1%)	3 (17.7%)	
**Grade of recommendation**, ***n*** **(%)**			0.089
Strong positive	0 (0%)	5 (29.4%)	
Weak positive	11 (78.6%)	9 (52.9%)	
Weak negative	3 (21.4%)	3 (17.7%)	
Strong negative	0 (0%)	0 (0%)	
**Summary of findings table which guided the recommendation**, ***n*** **(%)**			0.798
Summary of findings table n. 1	0 (0%)	0 (0%)	
Summary of findings table n. 2	0 (0%)	0 (0%)	
Summary of findings table n. 3	1 (7.1%)	3 (17.7%)	
Summary of findings table n. 4	1 (7.1%)	1 (5.9%)	
Two or more Summaries of Findings table	12 (85.7%)	13 (76.5%)	
None	0 (0%)	0 (0%)	
**Quality of evidence of the outcome considered the most important**, ***n*** **(%)**			0.884
High	9 (64.3%)	9 (52.9%)	
Moderate	3 (21.4%)	5 (29.4%)	
Low	2 (14.3%)	3 (17.7%)	
Very low	0 (0%)	0 (0%)	
**Criteria related to the summary of findings table which guided the choice (multiple answers allowed)**, ***n*** **(%)**			
N° of studies included	3 (21.4%)	2 (11.8%)	0.636
N° of patients included	8 (57.1%)	5 (29.4%)	0.119
Quality of the evidence	12 (85.7%)	13 (76.5%)	0.664
The benefit-to-risk ratio	10 (71.4%)	16 (94.1%)	0.148
Supplementary analyses	4 (28.6%)	5 (29.4%)	1.000
Evidence published in languages other than English	2 (14.3%)	0 (0%)	0.196
Personal experience or knowledge of the literature in the field	3 (21.4%)	1 (5.9%)	0.304
**Additional information requested in case of overlapping meta-analyses (multiple answers allowed)**, ***n*** **(%)**			
Results of the largest RCT	6 (42.9%)	3 (17.7%)	0.233
Heterogeneity / consistency	11 (78.6%)	9 (52.9%)	0.138
Included studies within each systematic review	5 (35.7%)	13 (76.5%)	0.022
Abstracts of each review	2 (14.3%)	2 (11.8%)	1
Directness	0 (0%)	0 (0%)	-
Details of search strategies	5 (35.7%)	5 (29.4%)	1
Limitations in primary studies	6 (42.9%)	8 (47.1%)	0.815
Risk of bias in each review	8 (57.1%)	13 (76.5%)	0.441
Full text of each review	0 (0%)	2 (11.8%)	0.488
Differences in PICOs	6 (42.9%)	12 (70.6%)	0.119
Details of methods used to combine studies	5 (35.7%)	9 (52.9%)	0.337
**Belief that the actual form of the summary of findings table captures differences across overlapping systematic reviews**, ***n*** **(%)**			0.290
Yes	2 (14.3%)	0 (0%)	
No	5 (35.7%)	9 (52.9%)	
Mixed feeling	7 (50.0%)	8 (47.1%)	

We published raw data from this study on the Open Science Framework; the raw data table is available on the following link: https://osf.io/qsbyh/.

## Discussion

We presented different SoF tables from overlapping SRs to methodologists and clinicians, who were somewhat familiar or experts with GRADE methodology and the development of SoF tables. Even though they addressed the same clinical question, i.e., systemic thrombolysis for intermediate-risk pulmonary embolism, the SoF tables reported results that were contradictory to some extent. Nevertheless, based on the quality of the SRs, participants were able to identify a recommendation-making process, giving preference to high-quality reviews. When faced with discordant evidence, they were more likely to formulate a weak recommendation. Additional information relevant to participants for supporting the clinical decision-making process were risk of bias, heterogeneity, and information about included studies in discordant SRs.

In 1997, the question of interpreting discordant results from similar SRs was first addressed (Jadad et al., 1997). It has been documented that escalation in the number of SRs increases the likelihood of finding conflicting evidence (Lucenteforte et al., 2015; Riva et al., 2018). These disputes between different SRs on the same topic may lead to the dissemination of inconsistent recommendations, slowing the transfer of research evidence into practice.

OSRs can easily identify cases of overlapping and discordant reviews. However, a recent analysis by Lunny and colleagues of 50 randomly sampled overviews showed that only a minority of them reported methods for handling overlapping reviews and discordant results and/or conclusions (Lunny et al., 2020). Another challenge for information users is also to appraise whether some of the discordant SRs meet the criteria of a SRas one or more may not be systematic in part or at all (Dettori and Norvell, 2020). Several typologies of literature reviews have been proposed (Par et al., 2015; Munn et al., 2018), contributing to different definitions of SR over time and across fields. The lack of a consensual definition of an SR and agreement on core methodological elements (e.g., identification of all relevant records, selection of eligible studies, assessment of the risk of bias, qualitative synthesis of the included studies, and meta-analyses when possible) can challenge an explicit and reproducible selection of SRs by researchers (Krnic Martinic et al., 2019).

This survey offers preliminary insights on concepts and practices among reviewers and health professionals who read SRs, or use them in the context of recommendations development when facing multiple sources of evidence. Based on our findings, we suggest that the structure of SoF tables should be complemented with a dedicated module of information to present users with details about overlapping and potentially discordant SRs. The purpose of such a tool would be to inform the reporting of overlapping evidence syntheses rather than provide a method to identify or classify discordant evidence. It could, however, be also used by reviewers, guideline developers and journal editors to plan for a comprehensive appraisal of evidence syntheses in situations where it is important to consider overlapping sources and conflicting results.

Using a new type of SoF table for OSRs, as a tool that would help collate the essential information about overlapping and potentially discordant reviews is likely to support clinical decision-making and more transparent guideline recommendations. As Bobbio and colleagues wrote in 1991 in a seminal paper in the Lancet about optimal reporting of trial results, “*the key question in most therapeutic controversies is not whether the proposed treatment is effective or not, but how data should be presented in order to allow physicians and patients to decide for themselves*” (Bobbio et al., 1994). Overall, a broader appreciation of the need for reporting findings from multiple sources, particularly when they reach high-quality thresholds, would be a welcome evolution in evidence synthesis science.

Our study has several potential limitations. There is no consensus definition of an opinion leader, which might have hampered our selection of experts that assessed the SoF tables. We used snowball sampling for the recruitment of participants. Such sampling is frequently used as a form of non-random sampling where a high external validity is not sought as prominent feature of a study (Parker et al., 2019). The initial sample relies on personal contacts. Thus, with this sampling strategy, we cannot generalize our results. In our study, we compared the responses of clinicians and methodologists. As the numbers of participants in each group were small, any difference should be interpreted as hypothesis-generating. Our study targeted a single medical question, namely thrombolytic therapy for intermediate-risk pulmonary embolism, again limiting external validity. Familiarity with the topic could have influenced the responses of some participants.

In conclusion, when faced with multiple controversial SR results, the type and completeness of reported information in SoF tables could affect experts' ability to make recommendations. Therefore, developers of the SoF table should consider collating the critical information from overlapping and potentially discordant reviews.

## Data Availability Statement

The datasets presented in this study can be found in online repositories. The names of the repository/repositories and accession number(s) can be found at: we published raw data on the Open Science Framework, and made it public. The raw data table is available on the following link: https://osf.io/qsbyh/.

## Ethics Statement

The study protocol was approved by the Ethics Committee of the University of Split School of Medicine (Approval no. 2181-198-03-04-17-0064). The patients/participants provided their written informed consent to participate in this study.

## Author Contributions

All authors study design, data analysis, data interpretation, writing of the manuscript, and approval of the final version of the manuscript.

## Funding

This work was supported by the Italian Ministry of Health (Giovani Ricercatori GR-2011-02348048) and by Regione Lombardia (R.L. d.g.r. n. IX/4662, 9/01/2013).

## Conflict of Interest

The authors declare that the research was conducted in the absence of any commercial or financial relationships that could be construed as a potential conflict of interest.

## Publisher's Note

All claims expressed in this article are solely those of the authors and do not necessarily represent those of their affiliated organizations, or those of the publisher, the editors and the reviewers. Any product that may be evaluated in this article, or claim that may be made by its manufacturer, is not guaranteed or endorsed by the publisher.
