# Supplementary material for: Mitigating Disputes Originated by Multiple Discordant Systematic Reviews and Meta-Analyses: A Survey of Methodologists and Clinicians
Source: Front Res Metr Anal. 2022 Apr 15;7:849019. doi: 10.3389/frma.2022.849019 (PMC9051432; doi:10.3389/frma.2022.849019)
Supplement: Supplementary file 1 [file Table_1.DOCX]

**Appendix 1. Summary of findings (SoF) tables from four published systematic reviews about thrombolytic therapy for patients with pulmonary embolism who have an intermediate risk of death**

**1. SoF from a systematic review of Chen et al.** [1]


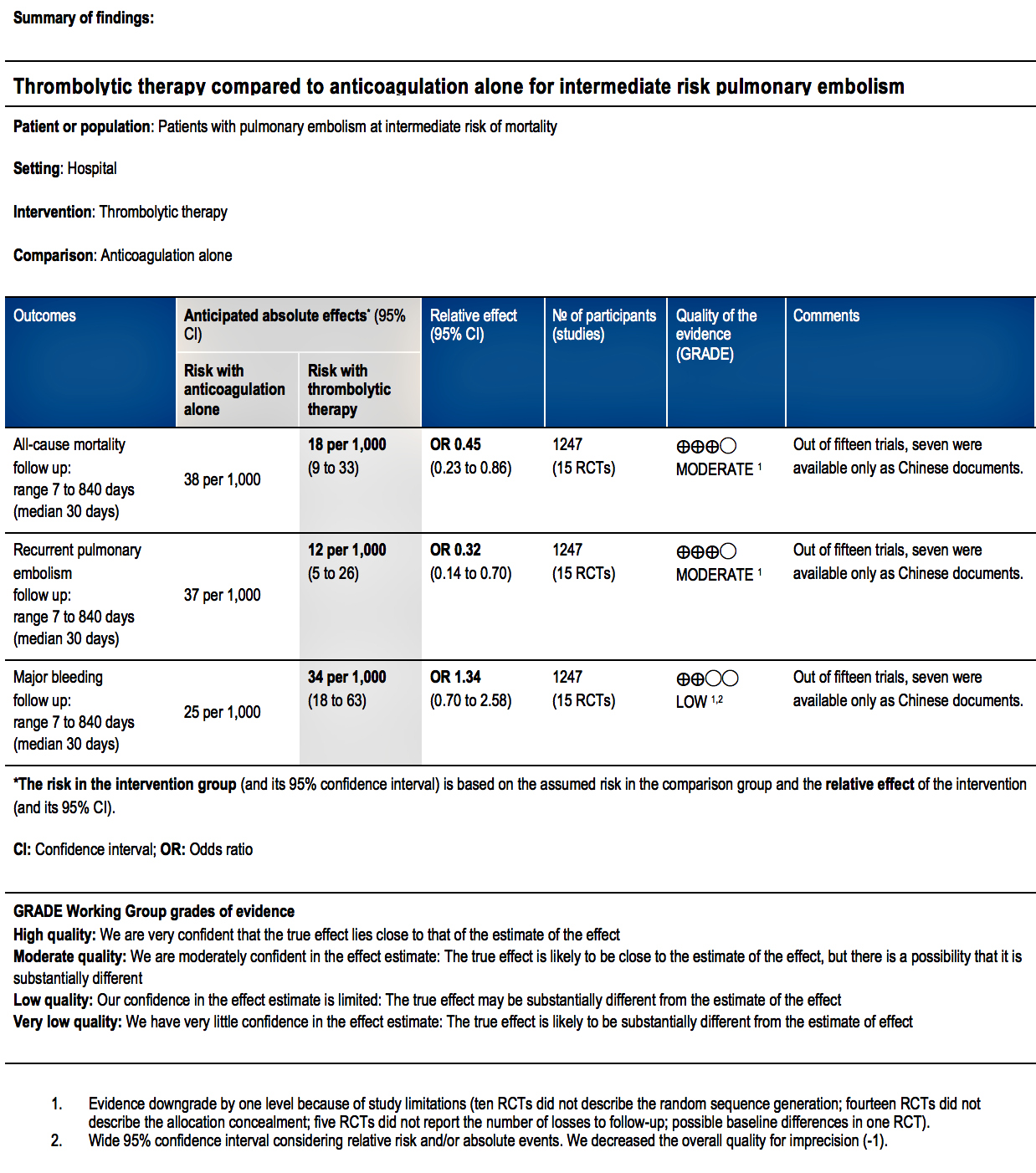


**2. SoF from a systematic review of Chatterjee et al.** [2]


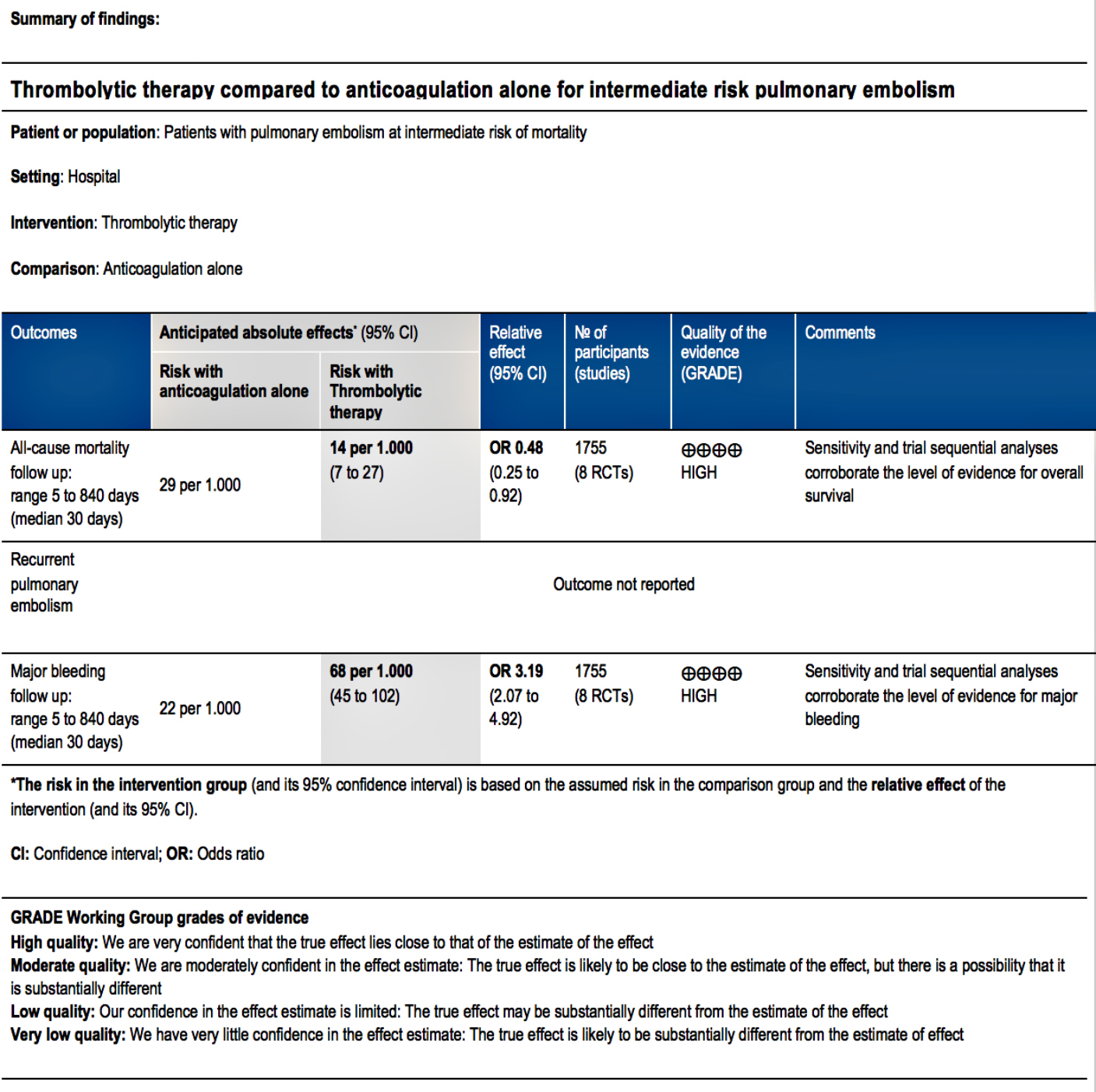


**3. SoF from a systematic review of Hao et al.** [3]


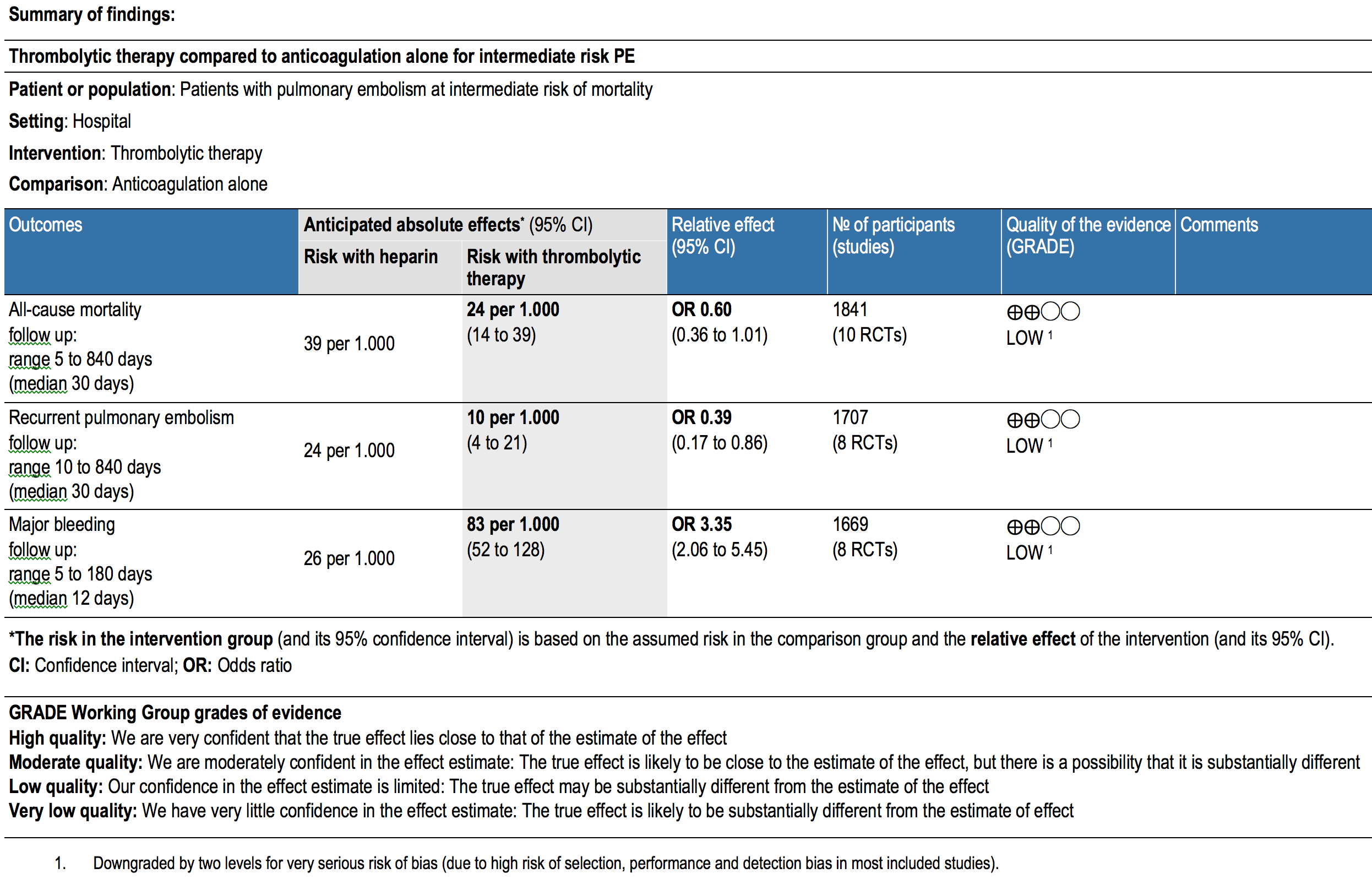


**4. SoF from a systematic review of Gao et al.** [4]


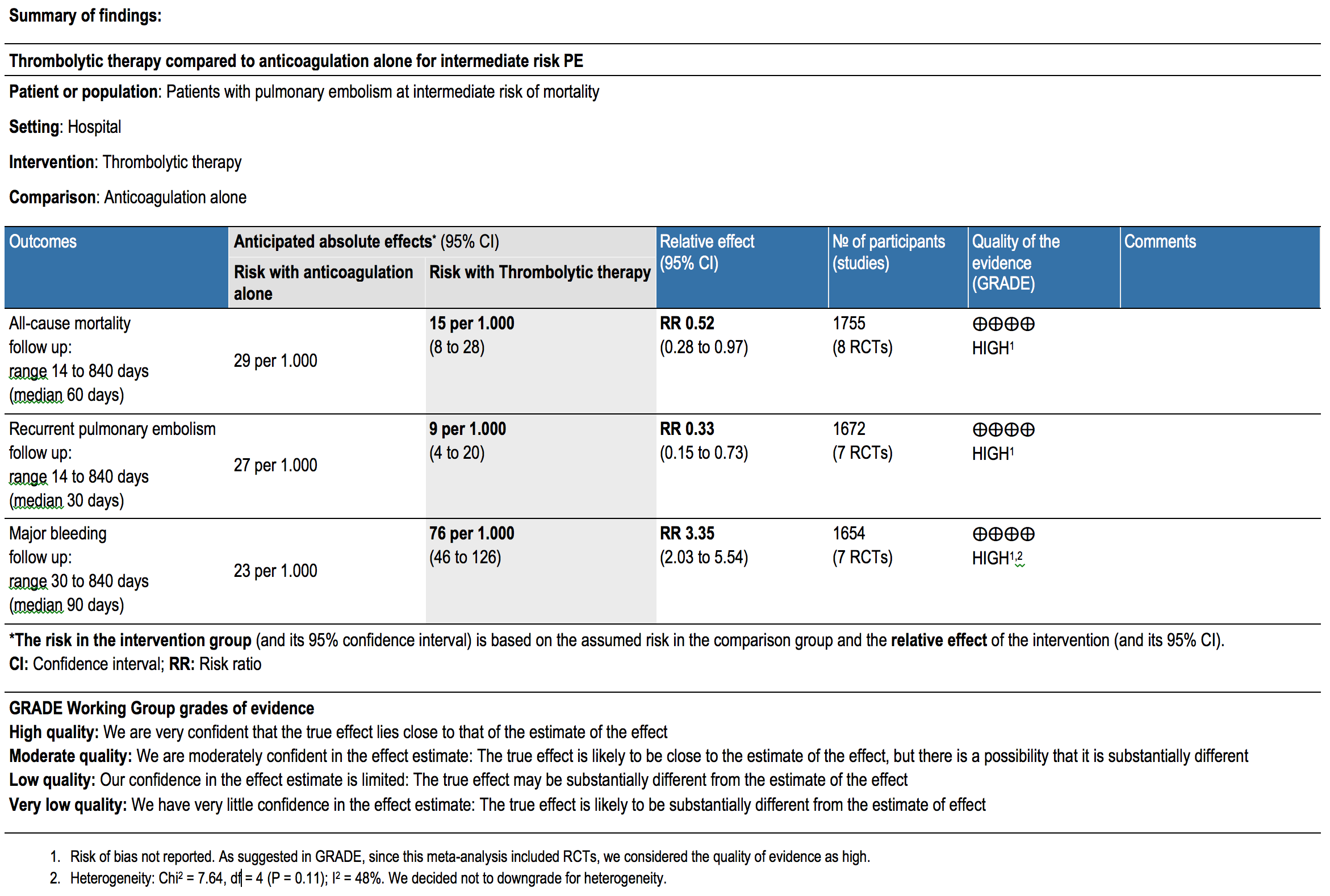


**References**

[1] Chen H, Ren C. Thrombolysis versus anticoagulation for the initial treatment of moderate pulmonary embolism: a meta-analysis of randomized controlled trials. Respir Care. 2014;59:1880-7.

[2] Chatterjee S, Chakraborty A, Weinberg I, Kadakia M, Wilensky RL, Sardar P, et al. Thrombolysis for pulmonary embolism and risk of all-cause mortality, major bleeding, and intracranial hemorrhage: a meta-analysis. JAMA. 2014;311:2414-21.

[3] Hao Q, Dong BR, Yue J, Wu T, Liu GJ. Thrombolytic therapy for pulmonary embolism. Cochrane Database Syst Rev. 2015:CD004437.

[4] Gao GY, Yang P, Liu M, Ding M, Liu GH, Tong YL, et al. Thrombolysis for acute intermediate-risk pulmonary embolism: A meta-analysis. Thromb Res. 2015;136:932-7.
