# Supplementary material for: Mitigating Disputes Originated by Multiple Discordant Systematic Reviews and Meta-Analyses: A Survey of Methodologists and Clinicians
Source: Front Res Metr Anal. 2022 Apr 15;7:849019. doi: 10.3389/frma.2022.849019 (PMC9051432; doi:10.3389/frma.2022.849019)
Supplement: Supplementary file 2 [file Table_2.DOCX]

**Appendix 2. Survey used in this study**

*Q1 Would you be willing to participate?*

Yes

No

*Q2 What is your gender?*

Male

Female

*Q3 What is your age?*

up to 30

31 to 40

41 to 50

51 to 60

61 to 70

71 to 80

81 or older

*Q4 In what country do you work?*

_________________

*Q5 Which term best describes your role?*

Clinician

Methodologist

Policy maker

Patients’ representative

Other

*Q6 How familiar are you with Summary of Findings (SoF) tables?*

Not familiar at all (I've never used them)

Very little familiar (I've only used them once)

A bit familiar (I've used them more than once, but I still need help when creating one)

Somewhat familiar (I've used them on several occasions but still need help on some issues)

Very familiar (I've used them on several occasions and/or I can help others create them)

Expert (I am involved in GRADE methods and I can teach others how to create SoF tables)

*Q7 Based on this evidence, please provide your recommendation: “in patients with intermediate risk pulmonary embolism, thrombolytic therapy compared with anticoagulation alone is”*

Recommended

Suggested

Might be suggested

Not recommended

*Q8 The grade for this recommendation is:*

Strong positive

Weak positive

Weak negative

Strong negative

*Q9 Which SoF guided your decision?*

Summary of Findings 1

Summary of Findings 2

Summary of Findings 3

Summary of Findings 4

Two or more Summaries of Findings

None

*Q10 The quality of evidence of the outcome that you consider to be the most important is:*

High

Moderate

Low

Very low

*Q11 Which criteria related to the Summary of Findings guided your choice?*

N° of studies included

N° of patients included

Quality of the evidence

The benefit-to-risk ratio

Supplementary analyses (e.g. sensitivity, trial sequential analysis mentioned in Summary of Findings 2)

Evidence published in languages other than English

My personal knowledge of the literature in the field or experience (criterion not related to the Summary of Findings)

*Q12 Overlapping meta-analyses can often be confusing because they may reach different conclusions. In such cases, which approach could be the most effective in supporting guideline development group decisions? Please order the statements by relevance, the most relevant=1.*

All systematic reviews, including redundant reviews

High-quality systematic reviews, excluding redundant

reviews

One systematic review, top ranked for quality

Key reviews representing discordant results, excluding

redundant reviews

The most recently updated systematic review

*Q13 In case of overlapping meta-analyses, on top of SoFs, which additional information would you like to have?*

Results of the largest RCT

Heterogeneity / consistency

Included studies within each systematic review

Abstracts of each review

Directness

Details of search strategies

Limitations in primary studies

Risk of bias (measured with AMSTAR or ROBIS) in each review

Full text of each review

Diferences in PICOs

Details of methods used to combine studies

Other (please specify)

*Q14 Does the actual form of the Summary of Findings Table captures differences across overlapping systematic reviews?*

Yes

No

Mixed feeling

*Q15 Do you have any suggestions to improve the Summary of Findings template in case of overlapping reviews?*

_________________

*Q16 If you would like to participate in another round of this survey, focussed on solutions to overlapping and discordant evidence in Summary of Findings, please give us your email.*

Yes

No, thanks, one was enough!

Your contact email: ________________________
